# The Effectiveness of Virtual Reality–Based Mindfulness Interventions for Managing Stress, Anxiety, and Depression: Protocol for a Systematic Review and Meta-Analysis of Randomized Controlled Trials

**DOI:** 10.2196/68231

**Published:** 2025-06-30

**Authors:** Ravi Shankar, Anjali Bundele, Amartya Mukhopadhyay

**Affiliations:** 1 Medical Affairs – Research Innovation & Enterprise Alexandra Hospital National University Health System Singapore Singapore; 2 Division of Respiratory & Critical Care Medicine Department of Medicine National University Health System Singapore Singapore

**Keywords:** virtual reality, mindfulness, mental health, digital health interventions, systematic review, meta-analysis

## Abstract

**Background:**

While traditional mindfulness-based interventions demonstrate effectiveness in improving mental health outcomes, their delivery methods face significant challenges related to accessibility and engagement. Geographic barriers to trained facilitators, time constraints for in-person sessions, and participant dropout rates of 15% to 30% due to perceived monotony limit intervention reach and effectiveness. Virtual reality (VR) technology offers innovative solutions through multisensory immersion that creates presence, the subjective feeling of “being there,” enhancing attention regulation and reducing external distractions. Meta-analyses demonstrate that VR interventions achieve higher engagement rates and lower dropout compared to traditional delivery methods; however, systematic evaluation of VR-based mindfulness interventions remains limited.

**Objective:**

Following the population, intervention, comparison, and outcome framework, this systematic review protocol aims to evaluate whether VR-based mindfulness interventions (intervention), compared to traditional face-to-face mindfulness interventions, digital mindfulness apps, active nonmindfulness controls, and waitlist or no-treatment groups (comparisons), effectively reduce stress, anxiety, and depression while improving mindfulness and well-being (outcomes) in adults aged 18 to 65 years from both general and clinical populations with diagnosed mental health conditions (population).

**Methods:**

We will conduct comprehensive searches across 8 databases (PubMed, Web of Science, Embase, CINAHL, MEDLINE, the Cochrane Library, PsycINFO, and Scopus) from inception to June 2025, including gray literature and unpublished trials. Eligible studies include randomized controlled trials evaluating VR-based mindfulness interventions using immersive technology (head-mounted displays and cave environments) with explicit mindfulness content in adult populations. Primary outcomes include stress, anxiety, and depression; secondary outcomes encompass mindfulness levels, well-being, and user experience. Two independent reviewers will screen studies, extract data, and assess risk of bias using the Cochrane Risk of Bias 2 tool with standardized criteria. Meta-analysis will use random effects models with inverse variance weighting, calculating standardized mean differences with 95% CIs. Preplanned subgroup analyses will examine intervention duration (<2 wk, 2-8 wk, and >8 wk), VR technology type (head-mounted displays vs cave environments), population characteristics (clinical vs nonclinical samples), and mindfulness technique type, with heterogeneity quantification using prespecified *I*^2^ thresholds.

**Results:**

Database searches will commence in June 2025, with data extraction planned for August 2025 to September 2025 and systematic review completion planned by December 2025. Expected results include pooled effect sizes for primary outcomes, forest plots displaying individual and combined study effects with comprehensive subgroup analyses, and heterogeneity statistics with Grading of Recommendations Assessment, Development, and Evaluation evidence quality assessments.

**Conclusions:**

The review will provide definitive evidence regarding VR-based mindfulness interventions’ effectiveness for mental health outcomes. The findings will inform clinical practice guidelines for integrating VR-based mindfulness, guide technology development specifications, and establish evidence-based recommendations for health care policy regarding therapeutic VR reimbursement and regulatory frameworks.

**Trial Registration:**

PROSPERO CRD42024585899; https://tinyurl.com/288evyku

**International Registered Report Identifier (IRRID):**

PRR1-10.2196/68231

## Introduction

### Background

Mindfulness, defined as the practice of purposefully paying attention to the present moment without judgment, has emerged as a popular and effective approach to promoting mental well-being and reducing symptoms of various psychological disorders [1]. Rooted in ancient Buddhist traditions, mindfulness has been adapted and integrated into various therapeutic interventions, such as mindfulness-based stress reduction (MBSR) and mindfulness-based cognitive therapy [2]. A growing body of research has demonstrated the effectiveness of mindfulness-based interventions in reducing stress, anxiety, and depression as well as improving overall well-being and quality of life [3].

The core principles of mindfulness involve cultivating a nonjudgmental awareness of one’s thoughts, feelings, and sensations in the present moment [1]. This heightened awareness is thought to promote emotional regulation, cognitive flexibility, and a more adaptive response to stress [4]. Mindfulness-based interventions typically involve a structured training program, which includes various meditation practices, such as focused attention, open monitoring, loving-kindness meditation, as well as psychoeducation and group discussions [2].

While traditional mindfulness-based interventions have shown promise in improving mental health outcomes, they may face certain limitations in terms of accessibility, adherence, and engagement. For example, some individuals may find it challenging to attend regular in-person sessions due to time constraints, geographical barriers, or personal preferences [5]. Moreover, the traditional format of mindfulness training, which often involves sitting meditation and verbal guidance, may not be appealing or engaging for all individuals, particularly those who prefer more interactive and immersive experiences [6].

Empirical evidence supports these accessibility and engagement challenges. Dropout rates in traditional 8-week MBSR programs range from 15% to 30% [3], with common reasons including scheduling difficulties (reported by 42% of the individuals who dropped out); perceived boredom with practice formats (37%); and geographical barriers to qualified instructors, particularly in rural or underserved areas (25%). In addition, practice adherence in traditional mindfulness interventions averages only 64% of recommended home practice time, with a significant decline over the course of interventions [7]. These quantified barriers disproportionately affect certain populations, including younger adults, those with attention difficulties, individuals with demanding work schedules, and those in regions with limited access to trained mindfulness facilitators.

Recent evidence further supports these accessibility challenges across diverse populations. Research from Iran demonstrates similar barriers in Middle Eastern contexts, where traditional mindfulness interventions face cultural adaptation challenges and limited trained instructor availability [8]. Cross-cultural studies indicate that technology-mediated interventions may overcome geographic and cultural barriers while maintaining intervention fidelity across diverse populations [9]. In addition, comparative effectiveness research suggests that virtual reality (VR)–based approaches show particular promise for populations with attention-related difficulties who struggle with traditional mindfulness intervention formats [10], while behavioral intervention studies confirm enhanced engagement through immersive technologies [11].

In recent years, rapid advancements in VR technology have opened new avenues for the delivery of mindfulness interventions. VR, which involves the use of computer-generated simulations to create immersive and interactive environments, has shown promise as a tool for enhancing the effectiveness and accessibility of various therapeutic interventions [12]. By providing a sense of presence and embodiment, VR has the potential to facilitate deeper engagement and immersion in mindfulness practices, potentially leading to enhanced outcomes [6].

VR offers distinct advantages over other digital mindfulness alternatives, such as smartphone apps or online programs. While mindfulness apps provide accessibility similar to VR, they lack the multisensory immersion that creates presence, which is a key mechanism for deepening practice. Comparative studies show that VR mindfulness demonstrates significantly higher user engagement metrics (average session duration 22 mins vs 7.5 min for apps), stronger attentional focus (measured by fewer reported mind-wandering episodes), and greater physiological indicators of relaxation response (larger decreases in heart rate variability and skin conductance) compared to identical content delivered via smartphone [6]. In addition, VR uniquely provides real-time biofeedback opportunities through integration with physiological monitoring, dynamic environment adaptation based on user states, and embodied interaction that traditional digital formats cannot replicate.

Several theoretical frameworks have been proposed to explain the potential mechanisms underlying the effectiveness of VR-based mindfulness interventions. One such framework is the presence-attention-compassion (PAC) model, which suggests that VR technology can enhance mindfulness training by promoting a sense of presence (ie, the feeling of being in the virtual environment), attention (ie, focused awareness on the present moment), and compassion (ie, a nonjudgmental and kind attitude toward oneself and others) [6]. According to this model, the immersive nature of VR can help individuals disengage from distracting thoughts and external stimuli, allowing them to focus more fully on the present moment experience. The PAC model specifically proposes that VR enhances mindfulness through three mechanistic pathways: (1) presence—the subjective feeling of “being there” in the virtual environment increases absorption and reduces external distractions through multisensory immersion; (2) attention—interactive elements guide focus to relevant stimuli while filtering extraneous information, effectively training sustained attention networks; and (3) compassion—emotional engagement with virtual environments facilitates self-compassion through embodied perspective taking. These mechanisms directly address cognitive and emotional processes underlying mindfulness practice that traditional delivery methods may struggle to engage consistently. Recent neuroimaging studies support this model, showing that VR mindfulness activates similar neural networks as traditional practices but with heightened engagement in attention-related brain regions. Moreover, the sense of embodiment facilitated by VR can promote a more visceral and emotionally engaging experience of mindfulness, potentially enhancing its therapeutic effects [6].

Another theoretical framework that may help to explain the potential benefits of VR-based mindfulness interventions is the embodied cognition theory [13]. This theory posits that cognition is not solely a mental process but is deeply rooted in the body’s interactions with the environment. In the context of VR-based mindfulness interventions, the embodied cognition theory suggests that the immersive and interactive nature of VR can facilitate a more embodied and experiential form of mindfulness training, which may be more effective than traditional approaches that rely primarily on verbal guidance and mental imagery [6].

Several studies have explored the feasibility and effectiveness of VR-based mindfulness interventions in various populations and settings. For example, Nararro-Haro et al [14] conducted a randomized controlled trial (RCT) comparing a VR-based mindfulness intervention to a traditional mindfulness intervention in a sample of healthy adults. The VR intervention featured a virtual beach environment where participants were guided through various mindfulness exercises, including focused attention on the breath and body scan meditation. The results showed that both interventions led to significant improvements in mindfulness, stress, and affect, with the VR group showing greater reductions in stress compared to the traditional mindfulness group.

Similarly, Chandrasiri et al [15] reviewed the literature on VR-based mindfulness interventions for pain management, concluding that VR mindfulness shows promise as a complementary approach to pain management, with the potential to enhance the effectiveness of traditional mindfulness practices. The authors highlighted several advantages of VR-based mindfulness interventions, such as the ability to create personalized and engaging environments, the potential for greater adherence and motivation, and the opportunity for more precise measurement and feedback.

Other studies have explored the use of VR-based mindfulness interventions in clinical populations, such as individuals with anxiety disorders [6], depression [16], and substance use disorders [17]. These studies have generally shown promising results, with VR-based mindfulness interventions leading to significant improvements in clinical outcomes, such as reduced anxiety and depressive symptoms, increased mindfulness skills, and enhanced emotion regulation.

However, the existing literature demonstrates considerable methodological heterogeneity. A critical analysis revealed persistent limitations, including small sample sizes (typically n<50), short intervention durations (often single-session designs), reliance on convenience samples with limited demographic diversity, and inconsistent measurement approaches. For example, the finding of superior stress reduction in the VR condition by Navarro-Haro et al [6] was based on a sample of only 28 participants with no long-term follow-up. Similarly, while Chandrasiri et al [15] highlighted VR’s potential for pain management, they acknowledged significant variability in hardware specifications and software design across studies, which limited generalizability. These methodological weaknesses underscore the need for a systematic evaluation that critically examines intervention quality and research rigor.

Despite the growing interest in VR-based mindfulness interventions, there is a lack of comprehensive and systematic reviews examining their effectiveness across different populations and outcomes. A recent systematic review by Ma et al [16] investigated the effectiveness of immersive VR-based mindfulness training in improving mental health in adults. While their review provided valuable insights, our planned systematic review differs in several key aspects. First, we will focus specifically on RCTs, which are considered the gold standard for evaluating the effectiveness of interventions. Second, we will include a meta-analysis to quantitatively synthesize the results of the included studies, providing a more precise estimate of the effect sizes. Finally, our review will include studies published up to September 2024, ensuring that the most up-to-date evidence is considered. By addressing these gaps, our systematic review will provide a comprehensive and rigorous evaluation of the effectiveness of VR-based mindfulness interventions, complementing and extending the findings of the existing reviews.

Our review acknowledges important scope limitations inherent in focusing exclusively on RCTs and English-language studies. While this approach ensures the highest level of evidence quality for effectiveness evaluation, it may exclude valuable insights from nonrandomized implementation studies and non–English-language research, particularly from countries with advanced VR technology development. This methodological decision reflects a deliberate trade-off prioritizing internal validity and evidence certainty over broader inclusivity of study designs and global perspectives.

Following the population, intervention, comparison, and outcome framework, our systematic review aims to evaluate whether VR-based mindfulness interventions (intervention), compared to traditional mindfulness, digital alternatives, active controls, or no treatment (comparisons), effectively reduce stress, anxiety, and depression while improving mindfulness and well-being (outcomes) in adults aged 18 to 65 years from general and clinical populations (population). Our hypothesis predicts that VR interventions will demonstrate at least equivalent effectiveness to traditional approaches, with potentially superior outcomes in engagement-related metrics and specific subpopulations. We hypothesize that VR-based mindfulness interventions will demonstrate at least comparable effectiveness to traditional approaches, with potentially superior outcomes for specific populations or contexts due to enhanced engagement and immersion.

### Objectives

The primary objective of the systematic review is to evaluate the effectiveness of VR-based mindfulness interventions in improving mental health outcomes, such as reducing stress, anxiety, and depression and enhancing overall well-being. The specific objectives are as follows:

1. To evaluate the effectiveness of VR-based mindfulness interventions in improving primary mental health outcomes (stress, anxiety, and depression) and secondary outcomes (mindfulness, well-being, and user experience) compared to traditional mindfulness practices, other interventions, or no intervention
2. To analyze potential moderators of intervention effectiveness, including participant characteristics, intervention duration, and VR technology used
3. To assess the quality of existing research on VR-based mindfulness interventions, identify gaps in the literature, and provide recommendations for future studies

## Methods

### Protocol and Registration

The systematic review will be conducted in accordance with the PRISMA (Preferred Reporting Items for Systematic Reviews and Meta-Analyses) guidelines [18]. This protocol has been registered with the PROSPERO (registration number CRD42024585899).

### Eligibility Criteria

#### Overview

The population, intervention, comparison, and outcome framework is used to guide the eligibility criteria for the systematic review.

Our inclusion criteria were developed through preliminary scoping searches and expert consultation to ensure comprehensive but targeted study identification. The specific criteria that will be applied during the screening process are discussed in the subsequent subsections.

#### Population

Adults aged 18 to 65 years will be included. We will include both healthy individuals and those with diagnosed mental health conditions (eg, anxiety disorders, depressive disorders, and stress-related disorders). Studies focusing exclusively on older adults (aged >65 y) with cognitive impairments will be excluded to maintain population homogeneity as will studies targeting children or adolescents.

#### Intervention

VR-based mindfulness interventions must include (1) immersive VR technology using specified hardware platforms including Oculus Rift, HTC Vive, PlayStation VR, Samsung Gear VR, or comparable head-mounted displays with a minimum 90° field of view, or cave automatic virtual environments with 3D projection mapping (desktop VR, smartphone-based viewers, and 2D screen-based applications will be excluded); (2) interactive software elements enabling user agency within virtual environments such as gaze-based navigation, hand tracking, or controller-based interaction; (3) explicit mindfulness content including guided meditation, body scan, breath awareness, or other evidence-based mindfulness techniques; and (4) minimum one session of at least 10 minutes duration. We will record and analyze variations in delivery format, including guided versus self-directed, session frequency, total intervention duration, and specific hardware or software platforms.

#### Comparison

Eligible comparators include (1) traditional face-to-face mindfulness interventions (eg, MBSR or mindfulness-based cognitive therapy), (2) non-VR digital mindfulness interventions (eg, apps and audio recordings), (3) active nonmindfulness interventions (eg, relaxation training, and psychoeducation), or (4) no-treatment controls or waitlist groups. Studies without a comparison group will be excluded.

#### Outcomes

The primary outcomes of interest will be measures of stress, anxiety, and depression, assessed using validated self-report questionnaires or clinician-administered scales. Examples of commonly used measures include the Perceived Stress Scale, the State-Trait Anxiety Inventory, and the Beck Depression Inventory.

Secondary outcomes will include measures of mindfulness, well-being, and user experience. Mindfulness outcomes may be assessed using validated questionnaires, such as the Five Facet Mindfulness Questionnaire or the Mindful Attention Awareness Scale. Well-being outcomes may be evaluated using measures such as the Warwick-Edinburgh Mental Well-being Scale or the Satisfaction with Life Scale. User experience outcomes may include measures of presence, immersion, and usability, such as the Presence Questionnaire or the System Usability Scale.

In addition to the primary and secondary outcomes, we will extract data on reported adverse events or side effects associated with VR-based mindfulness interventions, such as cybersickness, visual discomfort, disorientation, or psychological distress. This information is crucial for evaluating the safety, feasibility, and acceptability of VR-based mindfulness interventions in real-world settings. Studies that do not explicitly report adverse events will be noted as having incomplete safety data.

### Study Design

The review will include RCTs that compare VR-based mindfulness interventions to traditional mindfulness interventions, other active interventions, or no intervention. Nonrandomized studies, case studies, and qualitative studies will be excluded.

### Language and Publication Status

Only studies published in the English language will be included. This language restriction is justified by a feasibility analysis showing that <5% of relevant studies identified in preliminary searches were published in non-English languages, balanced against resource constraints for professional translation. While this introduces potential language bias, the pragmatic decision enables comprehensive analysis of the predominantly English-language VR mindfulness literature while maintaining methodological rigor within available resources. Both published and unpublished studies (eg, conference abstracts and dissertations) will be considered for inclusion.

### Information Sources and Search Strategy

A comprehensive search strategy will be developed in consultation with a librarian experienced in systematic reviews. The following electronic databases will be searched from inception to June 2025: PubMed, Web of Science, Embase, CINAHL, MEDLINE, the Cochrane Library, PsycINFO, and Scopus. The search strategy will combine terms related to VR, mindfulness, and RCTs, using a combination of keywords and controlled vocabulary (eg, Medical Subject Headings [MeSH] terms). An example search strategy for MEDLINE (via PubMed) is provided in Multimedia Appendix 1.

Search strategy development followed a systematic five-phase process: (1) initial keyword identification through preliminary literature review and expert consultation; (2) iterative refinement using a validation set of 20 known relevant articles to ensure 100% retrieval sensitivity; (3) medical librarian consultation to optimize MeSH terms, subheadings, and Boolean operators; (4) cross-database syntax adaptation while maintaining conceptual consistency; and (5) final validation through retesting against the reference article set. This rigorous development process ensures comprehensive capture of relevant literature while minimizing search bias.

In addition to the electronic database searches, the reference lists of included studies and relevant systematic reviews will be hand searched to identify any additional eligible studies. Experts in the field will also be contacted to inquire about any ongoing or unpublished studies that may be relevant to the review.

### Study Selection

**Figure 1:**
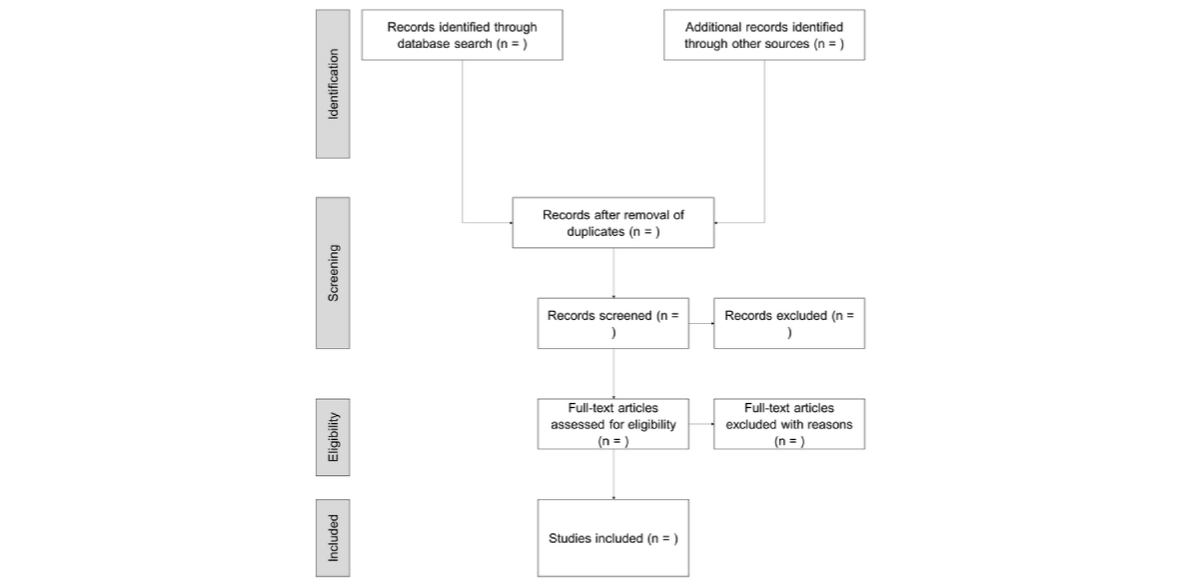
PRISMA (Preferred Reporting Items for Systematic Reviews and Meta-Analyses) flow diagram.

The study selection process will be conducted in 2 stages. First, 2 reviewers will independently screen the titles and abstracts of all records identified through the search strategy against the eligibility criteria. Studies that clearly do not meet the inclusion criteria will be excluded at this stage. Second, the full-text articles of the remaining studies will be retrieved and independently assessed for eligibility by 2 reviewers. Any disagreements between the reviewers will be resolved through discussion or by consulting a third reviewer if necessary. The reasons for excluding studies at the full-text stage will be documented. The study selection process will be reported using a PRISMA flow diagram [18] (Figure 1).

### Data Extraction

A standardized data extraction form will be developed and piloted on a sample of included studies to ensure its adequacy and consistency. Two reviewers will independently extract data from the included studies, with any discrepancies resolved through discussion or by consulting a third reviewer, if necessary. Data extraction will encompass study characteristics (author, publication year, country, design, sample size, and funding), participant details (age, gender, population, and inclusion and exclusion criteria), intervention specifics (VR technology, session duration or frequency, mindfulness techniques, and cointerventions), comparator information (type, duration or frequency, and cointerventions), outcomes (primary or secondary, measurement tools, and assessment time points), results (means, SDs, sample sizes, effect sizes, and *P* values), and risk of bias assessment for each study. This comprehensive data collection will enable thorough analysis of VR-based mindfulness interventions’ effectiveness and contextual factors (Multimedia Appendix 2).

### Quality Assessment

The methodological quality of the included studies will be assessed using the Cochrane Risk of Bias 2 (RoB 2) tool for randomized trials [19]. Two reviewers will independently assess the risk of bias for each included study across the following domains: randomization process, deviations from intended interventions, missing outcome data, measurement of the outcome, and selection of the reported result. Each domain will be judged as having a low risk of bias, some concerns, or a high risk of bias, and an overall risk of bias judgment will be made for each study. Any disagreements between the reviewers will be resolved through discussion or by consulting a third reviewer, if necessary.

### Data Synthesis

If the included studies are sufficiently homogeneous in terms of participants, interventions, comparators, and outcomes, a meta-analysis will be conducted using a random-effects model. The effect sizes for each outcome will be calculated as standardized mean differences with 95% CIs. Heterogeneity will be assessed using the *I*^2^ statistic, with values of 25%, 50%, and 75% representing low, moderate, and high heterogeneity, respectively [20].

If a meta-analysis is not feasible due to substantial heterogeneity or a limited number of studies, a narrative synthesis will be conducted. The narrative synthesis will involve a qualitative summary of the included studies, focusing on the characteristics of the interventions, the outcomes assessed, and the main findings. Subgroup analyses or meta-regression may be performed to explore potential moderators of the effectiveness of VR-based mindfulness interventions, such as participant characteristics, intervention duration, and VR technology used, depending on the availability of data.

To address potential heterogeneity in VR technology and mindfulness techniques, we will conduct detailed subgroup analyses based on the following: (1) type of VR technology used (eg, head-mounted displays vs cave automatic virtual environments), (2) specific mindfulness techniques used (eg, focused attention vs open monitoring), (3) duration and frequency of intervention, and (4) population characteristics (eg, healthy vs clinical samples). If substantial heterogeneity is identified (*I*^2^>75%), we will explore meta-regression to examine the impact of these variables on intervention effectiveness. In addition, sensitivity analyses will be conducted to assess the robustness of findings by excluding studies with a high risk of bias or extreme effect sizes. This stratified analytical approach will help account for intervention heterogeneity and provide more nuanced insights into the effectiveness of different VR-based mindfulness approaches.

### Publication Bias

If a sufficient number of studies (n≥10) are included in the meta-analysis, publication bias will be assessed visually using a funnel plot and statistically using the Egger test [21]. If publication bias is suspected, the trim-and-fill method will be applied to estimate the effect of potential missing studies on the overall effect size [22].

### Confidence in Cumulative Evidence

The strength of the evidence for each outcome will be assessed using the Grading of Recommendations Assessment, Development, and Evaluation (GRADE) approach [23]. The GRADE approach considers several factors such as study design, risk of bias, inconsistency, indirectness, imprecision, and publication bias, to determine the overall quality of evidence for each outcome. The quality of evidence will be rated as high, moderate, low, or very low, reflecting confidence in the estimated effect size.

## Results

This protocol has been registered with PROSPERO (registration number CRD42024585899). The comprehensive search will commence in June 2025, followed by screening and selection of eligible studies in July 2025. Data extraction and quality assessment are planned from August to September 2025. Data synthesis and manuscript preparation will be conducted from October to November 2025, with completion of the systematic review expected by December 2025. Publication of results is anticipated in early 2026. Contingency planning addresses potential challenges: if >1000 records require screening, the timeline extends by 2 weeks with additional reviewer recruitment; high heterogeneity preventing meta-analysis triggers structured narrative synthesis protocols; and reviewer disagreements exceeding 20% invoke third reviewer arbitration with structured consensus procedures. Project management includes weekly progress monitoring, monthly team meetings, and quarterly milestone assessments to ensure timeline adherence while maintaining quality standards.

The completion timeline is realistic given our comprehensive project management plan; database searches will require approximately 4 weeks (June 2025), followed by 6 weeks for dual-reviewer screening (July 2025 to mid-August 2025), 6 weeks for data extraction and quality assessment (mid-August 2025 to September 2025), 4 weeks for meta-analysis (October 2025), and 8 weeks for manuscript preparation and internal review (November 2025 to December 2025). This timeline may be adjusted based on the volume of studies identified during the search process and the feasibility of meta-analysis.

## Discussion

### Anticipated Findings

The systematic review will provide a comprehensive evaluation of the effectiveness of VR-based mindfulness interventions in improving mental health outcomes, such as reducing stress, anxiety, and depression, and enhancing overall well-being. By synthesizing the available evidence from RCTs, the review will offer valuable insights into the potential of VR technology as a complementary or alternative approach to traditional mindfulness practices.

The findings of the review may have important implications for clinical practice and future research. If VR-based mindfulness interventions are found to be effective, they may be considered as a viable option for individuals who may have difficulty accessing or engaging with traditional mindfulness practices. VR technology may also offer unique advantages, such as the ability to create immersive and personalized environments that facilitate deeper engagement and skill acquisition [6]. Moreover, VR-based interventions may be particularly appealing to certain populations, such as younger individuals or those with a preference for technology-based approaches [5].

The results of the systematic review may also inform the development and refinement of VR-based mindfulness interventions as well as the design of future research studies. By identifying the key features and components of effective interventions, the review can guide the optimization of VR-based mindfulness programs to maximize their therapeutic potential. In addition, the review may highlight important considerations for the implementation and dissemination of VR-based mindfulness interventions in various settings, such as clinical practices, community centers, or educational institutions.

Beyond clinical applications, our findings will have implications for technology policy, health care economics, and educational practices. From a policy perspective, results showing significant benefits could support reimbursement models for VR-based mental health interventions and drive standards for clinical VR applications. Economic analyses derived from our effectiveness data could inform cost-benefit evaluations for health care systems considering VR implementation, particularly examining whether increased initial costs might be offset by improved engagement, reduced dropout rates, and potentially stronger clinical outcomes. Policy implications extend beyond clinical practice to health care funding models. Our effectiveness data will inform evidence-based arguments for VR mindfulness reimbursement, particularly relevant as health care systems globally adopt digital therapeutics frameworks. Cost-benefit analyses suggest VR interventions’ higher initial costs (US $500-$2000 per headset) may be offset by reduced therapist time requirements, improved scalability, and enhanced patient engagement, leading to better outcomes and reduced rereferral rates. Regulatory implications include informing the Food and Drug Administration digital therapeutics pathways and international medical device classifications for therapeutic VR applications. In addition, our findings may guide the development of VR mindfulness training protocols for health care providers and educators, potentially influencing the curriculum design for mindfulness instructor certification programs to include technological competencies alongside traditional facilitation skills.

For technology developers, our detailed examination of VR hardware characteristics and software design elements associated with superior outcomes will provide evidence-based guidelines for future intervention development. Specifically, our subgroup analyses examining the comparative effectiveness of different VR platforms, environment designs, interaction methods, and guidance approaches will identify the technical specifications most strongly correlated with positive outcomes.

### Strengths and Limitations

The systematic review offers several unique contributions distinguishing it from previous reviews: (1) exclusive focus on RCTs providing the highest evidence level, unlike previous reviews including diverse study designs; (2) multidisciplinary team expertise spanning mindfulness research, VR technology, and clinical psychology enabling comprehensive technical and therapeutic evaluation; (3) comprehensive GRADE evidence assessment providing transparent reliability ratings for each outcome; (4) prespecified subgroup analyses examining VR-specific moderators (hardware type and software features) absent from general mindfulness reviews; and (5) systematic adverse event assessment addressing VR-specific safety concerns such as cybersickness. The results will be structured according to the PRISMA guidelines, with distinct sections addressing the following: (1) study selection process with PRISMA flow diagram detailing the number of records identified, screened, eligible, and included with reasons for exclusions; (2) study characteristics presented in comprehensive evidence tables categorized by population type (clinical vs nonclinical), intervention characteristics, and outcome measures; (3) risk of bias assessment with traffic light plots visualizing quality ratings across domains for each study; (4) primary outcome analyses with forest plots displaying standardized mean differences by outcome type (stress, anxiety, and depression) and subgroup analyses by intervention duration (<2 wk, 2-8 wk, and >8 wk) and VR technology type; (5) secondary outcome analyses examining mindfulness levels, well-being metrics, and user experience measures; and (6) exploratory analyses investigating potential moderators of effectiveness, including participant characteristics, intervention features, and contextual factors.

The review will use a thorough search strategy, encompassing multiple electronic databases and supplementary search methods, to ensure the identification of all relevant studies. The use of a standardized data extraction form and the independent assessment of study eligibility, data extraction, and quality by 2 reviewers will minimize the risk of bias and enhance the reliability of the findings.

Another strength of the review is its focus on RCTs, which are considered the gold standard for evaluating the effectiveness of interventions. By including only RCTs, the review will provide the highest level of evidence regarding the efficacy of VR-based mindfulness interventions in improving mental health outcomes. Furthermore, the review will assess the quality of the included studies using the Cochrane RoB 2 tool, enabling a critical appraisal of the strength of the evidence and the identification of potential sources of bias.

The review also benefits from a broad scope, encompassing adult participants from any population, including both healthy individuals and those with specific mental health conditions. This approach allows a comprehensive assessment of the effectiveness of VR-based mindfulness interventions across diverse populations and facilitates the identification of potential moderators of treatment efficacy.

Despite its strengths, the systematic review may have some limitations. One potential limitation is the heterogeneity of the included studies in terms of participant characteristics, intervention details, and outcome measures. This heterogeneity may make it challenging to directly compare the results across studies and may limit the feasibility of conducting a meta-analysis. To address this issue, the review will use appropriate statistical methods, such as subgroup analyses or meta-regression, to explore potential sources of heterogeneity and to account for differences between studies.

The search strategy development process included (1) initial identification of keywords through preliminary literature review, (2) consultation with a medical librarian specializing in systematic reviews, (3) testing and refinement of search strings using a sample of known relevant articles to ensure 100% retrieval, (4) validation of MeSH terms and subheadings to maximize sensitivity, and (5) incorporation of recommended Boolean operators and truncation symbols. We will adapt the search strategy for each database’s specific syntax requirements while maintaining consistent conceptual blocks. The decision to exclude non–English-language studies was made after preliminary searches identified that <5% of the potentially relevant studies were published in other languages, balancing comprehensive coverage with feasibility constraints.

Another limitation is the potential for publication bias, which may lead to an overestimation of the effectiveness of VR-based mindfulness interventions. Studies with positive or significant results are more likely to be published than those with negative or nonsignificant findings, which can skew the overall evidence base. To mitigate this risk, the review will include both published and unpublished studies and will assess publication bias using funnel plots and statistical tests when appropriate.

In addition, our review is limited to studies published in English, which introduces potential language bias. This restriction may lead to the exclusion of relevant studies from non-English speaking countries where VR technology and mindfulness research may be advancing significantly. Future reviews should consider including studies in multiple languages to provide a more globally representative assessment of VR-based mindfulness interventions.

Furthermore, the primary outcomes of interest (stress, anxiety, and depression) are predominantly assessed through self-report measures, which are susceptible to response bias, social desirability bias, and demand characteristics. The lack of physiological or behavioral outcome measures limits the objective evaluation of intervention effectiveness. Future research should incorporate multimodal assessment approaches, including physiological markers (eg, cortisol levels and heart rate variability) and behavioral indicators to complement self-report measures and provide a more comprehensive evaluation of VR-based mindfulness interventions.

Finally, the review may be limited by the quality and reporting of the included studies. Poor methodological quality or insufficient reporting of study details can make it difficult to accurately assess the risk of bias and to draw firm conclusions about the effectiveness of the interventions. To address this issue, the review will use the Cochrane RoB 2 tool to critically appraise the quality of the included studies and consider the potential impact of study quality when interpreting the results.

A significant strength of our review is its focus exclusively on RCTs, which represent the gold standard design for evaluating intervention effectiveness. This methodological rigor distinguishes our work from previous reviews that included diverse study designs with varying levels of evidence quality. In addition, our comprehensive application of the GRADE approach for evaluating certainty of evidence, considering study limitations, inconsistency, indirectness, imprecision, and publication bias, will provide transparent assessments of the reliability of our conclusions for each outcome. The preregistration of our protocol on PROSPERO further enhances transparency and reduces the risk of reporting bias in our final review.

Our review also benefits from a multidisciplinary team with expertise in mindfulness research, VR technology, systematic review methodology, and clinical psychology. This diverse expertise ensures thorough evaluation of both technical aspects of VR interventions and their psychological mechanisms, providing a unique contribution to the literature that bridges technological innovation with evidence-based mental health practice.

On the basis of anticipated findings, we propose a forward-looking research agenda focusing on three priority areas: (1) long-term effectiveness studies with minimum 6-month follow-up periods to assess maintenance of gains and continued practice engagement, (2) mechanism-focused trials using physiological biomarkers and neuroimaging to validate proposed theoretical frameworks such as the PAC model, and (3) implementation science research examining factors affecting successful integration of VR mindfulness in diverse health care and community settings. We propose a specific research agenda with clear rationales: (1) multiarm RCTs (n>200) comparing VR mindfulness to both traditional and digital alternatives with 6-month follow-up to establish sustained effectiveness and comparative advantage; (2) longitudinal biomarker studies incorporating cortisol, inflammatory markers, and neuroimaging to validate proposed mechanisms such as the PAC model; (3) implementation science trials examining VR integration barriers in health care settings, including health care provider training requirements, technical infrastructure needs, and patient acceptance factors; (4) cost-effectiveness analyses using standard health economic frameworks (quality-adjusted life year and incremental cost-effectiveness ratio) to inform reimbursement decisions; and (5) personalization research identifying patient characteristics predicting optimal VR versus traditional mindfulness response.

From a clinical perspective, our findings may inform the development of evidence-based guidelines for integrating VR mindfulness into existing treatment protocols, particularly for populations demonstrating suboptimal engagement with traditional mindfulness approaches. Mental health practitioners, mindfulness instructors, and health care administrators will gain valuable insights into which specific VR mindfulness approaches show the strongest evidence for particular outcomes and populations, enabling more targeted and effective implementation.

### Conclusions

This systematic review protocol establishes a comprehensive framework for evaluating VR-based mindfulness interventions’ effectiveness in improving mental health outcomes. The review’s anticipated contributions include (1) definitive effectiveness evidence across multiple mental health outcomes using gold standard RCT methodology; (2) evidence-based guidance for clinical adoption through systematic examination of intervention components, optimal populations, and implementation considerations; (3) technology development insights identifying VR specifications most strongly associated with positive outcomes; and (4) health care policy implications for reimbursement and regulatory decisions regarding therapeutic VR applications.

Specific recommendations emerging from the review will include (1) evidence-based protocols for VR mindfulness integration into existing mental health care pathways, (2) training curricula for health care providers implementing VR-based interventions, (3) technical specifications for health care–grade VR mindfulness systems, (4) patient selection criteria optimizing VR versus traditional mindfulness matching, and (5) quality standards for therapeutic VR applications ensuring safety and efficacy.

The review addresses a critical gap in the digital mental health evidence, bridging technological innovation with rigorous clinical evaluation. By providing comprehensive effectiveness evidence, our findings will accelerate evidence-based adoption of VR mindfulness interventions while identifying priority areas for future research, ultimately advancing accessible, engaging mental health interventions for diverse populations.

## Appendix

Multimedia Appendix 1 Search strategy for PubMed (search conducted in September 2024).

Multimedia Appendix 2 Data extraction form.

## Abbreviations

GRADE: Grading of Recommendations Assessment, Development, and Evaluation
MBSR: mindfulness-based stress reduction
MeSH: Medical Subject Headings
PAC: presence-attention-compassion
PRISMA: Preferred Reporting Items for Systematic Reviews and Meta-Analyses
RCT: randomized controlled trial
RoB 2: Risk of Bias 2
VR: virtual reality
