# Supplementary material for: The Effectiveness of Virtual Reality–Based Mindfulness Interventions for Managing Stress, Anxiety, and Depression: Protocol for a Systematic Review and Meta-Analysis of Randomized Controlled Trials
Source: JMIR Res Protoc. 2025 Jun 30;14:e68231. doi: 10.2196/68231 (PMC12260462; doi:10.2196/68231)
Supplement: Multimedia Appendix 1 [file resprot_v14i1e68231_app1.docx]

**Appendix A: Search Strategy for PubMed (Search conducted in September 2024)**

| **S.No.** | **Search Query** | **Records retrieved*** |
| --- | --- | --- |
| #1 | ('virtual reality' OR 'VR' OR 'head-mounted display' OR 'HMD' OR 'computer-generated' OR 'computer generated' OR 'immersive') | 523,782 |
| #2 | ('mindfulness' OR 'mindful*' OR 'mindfulness-based' OR 'mindfulness based' OR 'MBSR' OR 'MBCT' OR 'meditat*') | 37,695 |
| #3 | ('stress' OR 'anxiety' OR 'depression' OR 'well-being' OR 'wellbeing' OR 'user experience' OR 'presence' OR 'immersion' OR 'usability') | 12,675,197 |
| #4 | ('randomized controlled trial' OR 'controlled clinical trial' OR 'randomized' OR 'randomised' OR 'placebo' OR 'randomly' OR 'trial' OR 'groups') | 7,615,868 |
| #5 | #1 AND #2 AND #3 AND #4 | 330 |
| No limitations to language or date were applied. | |  |
