# Supplementary material for: The Effectiveness of Virtual Reality–Based Mindfulness Interventions for Managing Stress, Anxiety, and Depression: Protocol for a Systematic Review and Meta-Analysis of Randomized Controlled Trials
Source: JMIR Res Protoc. 2025 Jun 30;14:e68231. doi: 10.2196/68231 (PMC12260462; doi:10.2196/68231)
Supplement: Multimedia Appendix 2 [file resprot_v14i1e68231_app2.docx]

**Appendix B: Data Extraction Form**

| **Category** | **Data Items** |
| --- | --- |
| Study characteristics | First author, Year of publication, Country, Study design, Sample size, Funding source |
| Participant characteristics | Age (mean, SD, range), Gender (% female), Population (healthy, clinical), Inclusion/exclusion criteria |
| Intervention details | VR technology used, Duration of intervention, Frequency of sessions, Mindfulness techniques employed, Co-interventions |
| Comparator details | Type of comparator, Duration of comparator, Frequency of sessions, Co-interventions |
| Outcomes | Primary outcomes (stress, anxiety, depression): Measurement tools, Time points assessed; Secondary outcomes (mindfulness, well-being, user experience): Measurement tools, Time points assessed |
| Results | Means, standard deviations, and sample sizes for each outcome at each time point; Effect sizes (if reported); p-values (if reported) |
| Risk of bias assessment | Randomization process, Deviations from intended interventions, Missing outcome data, Measurement of the outcome, Selection of the reported result, Overall risk of bias judgment |
